# Academic training and clinical placement problems to achieve nursing competency

**Published:** 2015-01

**Authors:** NARJES RAHMATI SHARGHI, ALI ALAMI, SHAHLA KHOSRAVAN, MOHAMMAD REZA MANSOORIAN, ALI EKRAMI

**Affiliations:** 1 Research Student Committee, Gonabad University of Medical Sciences, Gonabad, Iran;; 2 Department of Health, Faculty of Public Health, Social Determinants of Health Research Centre, Gonabad University of Medical Sciences, Gonabad, Iran;; 3 Department of Community and Mental Health Nursing, Faculty of Nursing and Midwifery, Social Determinants of Health Research Centre, Gonabad University of Medical Sciences, Gonabad, Iran;; 4 Department of Community and Mental Health Nursing, Faculty of Nursing and Midwifery, Gonabad University of Medical Sciences, Gonabad, Iran;; 5 Students’ affairs Office, Cultural and students’ affairs Deputy, Gonabad University of Medical Sciences, Gonabad, Iran

**Keywords:** Academic training, Clinical, Achieve, Competency

## Abstract

**Introduction:** High quality of care is one of the requirements of nursing which depends on the nursing competency. In this connection, the aim of this research was to determine the problems related to the academic training (nursing' educational program) and clinical practice to achieve competency from the viewpoint of nurses, faculty members, and nursing students.

**Methods:** the study was an analytical cross-sectional one. The sample consisted of the academic staff, the third and the fourth year nursing students and nurses in practice. The instrument of the study was a two-part researcher-made questionnaire with 22 questions in the theoretical- clinical realm to assess  problems related to the theoretical and clinical teaching in nursing, and 23 questions to assess the clinical functions. The questionnaire was validated in terms of both face and content validity. Its reliability, using Cronbach's Alpha coefficient, was 0.72 in the theoretical-clinical and 0.73 in the clinical realm. Both descriptive and analytical statistics were used to analyze the data, using SPSS software.

**Results**: The results of this study indicated that from the participants’ viewpoints, the most important problems in the academic education for nursea to acquire competency were as follows: lack of academic research the clinical period (88.9%), no application of theoretical aspects of the nursing process in practice (85.6%), insufficient knowledgeable and professional educators (81.1%), the use of traditional routine-oriented methods on the wards (75.6%); also insufficient time for performance based on knowledge in relation to  the nurse's workload (86.5%), weakness and usefulness of scientific function encouragement systems in clinic (85.2%), and learnt theoretical subjects not coming into practice in clinical fields after graduation (75.6%).

**Conclusion: **Efforts to reduce the gap between the theoretical and practical (clinical function) knowledge in educational and work environment are required to improve the training of qualified nurses.

## Introduction

Nowadays, providing high quality nursing care is a requirement which depends on the nursing competency. Clinical nursing competency means competence and qualification in the areas of cognitive and psycho-physical, clinical skills, critical thinking, decision making and ability to enhance learning through academic knowledge and clinical experience, leading, finally, to standards and safe care (1).

The close relationship between clinical competency and the concepts of quality has a unique place in nursing profession as a practical profession. Some studies in Iran and other countries have shown that clinical competence of the nurses, and specially, the newly graduated nurses is far from the ideal situation (2, 3). Incompetent nurses in the clinical environment would be a danger to people’s health. Moreover, this leads to patients’ dissatisfaction (1). The process of developing professional competency in nursing begins from nursing school through formal academic programs, and develops by continuing educational programs in transition to workplace (on entry to practice). The role of nursing education is to improve the qualitative nursing practice (4).

So training competent nurses to preserve people’s health is consistently one of the goals of nursing education and this has drawn a greater attention to the subject of clinical qualification and competency (5, 6).

Nurse’s education, experience, professional development, independence, personal characteristics, motivation, and work satisfaction as well as the evaluation of the quality of nursing care have been identified as factors associated with nursing competency (6, 7). These factors were assessed in students by Mozingo et al. (1995). More practice of technical skills was introduced as an important factor in this study (8).

The role of competency in education has grown dramatically as health care employers and educators have identified the gap between education and practice. The gaps between educational preparation and actual practice were attributed to factors such as lack of funding to update the nursing curricula and a limited focus on teaching in academic health centres (9).

Cheraghi argues that there is a mutual relationship between knowledge and practice in nursing. Paradoxes in nursing knowledge and nursing practice which are seen along with diverging organizational structure reduce the ability of nurses in using professional knowledge and nursing skills (10).

Overall, the studies demonstrated the importance of clinical environment and academic nursing education in nursing competency. Therefore, the aim of this study was to assess problems to achieve competency from the viewpoints of nurses, faculty members, and nursing students. 

## Methods

This analytic, cross-sectional study was conducted in Gonabad University of Medical Sciences, using a census method. The sample consisted of 12 academic staff, 60 nursing students (the third and the fourth year), and 129 staff nurses working at the University hospitals. By a two-part researcher-made questionnaire the viewpoint of the participants on academic and clinical problems of competency in nursing training, in the academic teaching situation (theoretical- clinical realm) and practical function in hospital has been assessed . There were 22 questions in theoretical-clinical realm to assess problems related to the theoretical and clinical teaching in nursing, and 23 questions to assess the problems nurses and nursing graduates were involved in in real practice from the viewpoints of the participants. The response options for each question were on a 4-point scale, ranging from completely agree to completely disagree. The responses were collapsed to two groups, agree vs disagree, in data analysis. The questionnaire was validated in terms of both face and content validity. Its reliability, using Cronbach's Alpha coefficient, was 0.72 for the theoretical-clinical and 0.73 for the clinical realm. 

 This study was approved by the Student Research Committee of Gonabad University of Medical Sciences. After obtaining permission from the university vice chancellor for education, the ethics committee and heads of educational hospitals, the informed consent was obtained from all participants before delivery of the questionnaire. It was explained to all participants that the data obtained would remain confidential and the results would be presented in total. We used SPSS software (version 14) to analyse the data. The Chi-square test was used to determine the relationship among variables. p≤0.05 was considered significant. 

## Results

The participants’ mean age was 28.18±7.32 years, ranging from 20 to 51 years. Average number of the nursing graduates and employed staff was 8.27±6.53 years. The results related to academic and clinical problems regarding nursing competency are shown in Table 1. According this Table, the three groups of participants believed that in the academic teaching situation (theoretical-clinical realm), the theoretical content was in line with clinical needs (p=0.509) and was appropriately based on specific knowledge of nursing (p= 0.957). But nursing students stated that in every session of theoretical courses, the time allocated to teaching specific knowledge for nursing intervention, according nursing process, was not enough (i.e. Assessment, diagnosis, planning, intervention and evaluation). Another important theoretical and clinical problems were that academic research was not practiced in clinical areas (88.9%), theoretical aspects of the nursing process and not being applied in practice (85.6%), work of knowledgeable and professional educators often in faculties (81.1%), and educators following traditional routine-oriented ways in their nursing care in the ward (75.6%).

**Table 1:** Education realm’s (theoretical and practical) problems to achievement competency from Faculty member, students and nurses viewpoint

**Variables**	**Faculty member(n=12** **(**	**Nursing student(n=60)**	**Nurses(n=129)**	**Total(n=201)**	**p**
	**Agree (N%** **)**	**Agree (N%)**	**Agree (N%)**	**Agree (N%)**	
Non-use of research results in clinical wards	12(100.0)	55(91.7)	110(86.6)	177(88.9)	0.266
Failure to implement aspects of the nursing process in practice	12(100)	51(85)	109(84.5)	172(85.6)	0.340
Nurses with MSc. Or PhD degree teach in the Nursing faculties not in clinical ward	12(100.0)	50(83.3)	119(91.9)	181(91.9)	0.012
Educators following traditional routine-oriented ways in their nursing care on the wards	11(91.7)	48(80)	93(73.2)	152(76.4)	0.261
There is a gap between treatment and education systems	11(91.7)	43(71.7)	94(72.9)	141(73.6)	0.338
Educators use problem solving method in order to track students’ learning	5(41.7)	20(33.3)	48(40)	119(62)	0.661
Educators’ weaknesses in running scientific procedures based on theoretical courses in the internship environment	5(41.7)	31(51.7)	92(74.8)	128(65.6)	0.002
Teaching the theoretical subjects based on Specific knowledge of nursing	8(66.7)	39(65)	86(67.2)	133(66.5)	0.975
The amount of time devoted to holding internship with theoretical volume	5(41.7)	20(33.3)	51(39.5)	125(62.5)	0.687
The amount of time devoted to teaching special nursing knowledge	5(41.7)	16(26.7)	62(48.1)	83(41.3)	0.021
Relevance of theoretical course’s content and clinical needs	8(66.7)	29(48.3)	67(51.9)	104(51.7)	0.509
Effective and useful scientific relation between educators, nurses and doctors	3(25)	12(20)	34(26.4)	49(24.4)	0.638

* Chi-square test

The clinical realm (theoretical and practical) problems from the viewpoints of professors, students and nurses are shown in Table 2. According to this Table, the most important problem was emphasis on theoretical topics rather than practical aspects (p<0.001). The participants stated that the learnt theoretical subjects did not come into practice after graduation. Besides, there were no standard measures of the nursing care as well as relevant facilities on the wards. In addition, because of the nurses’ workload, there was not enough time for knowledge-based function.

**Table 2:** Clinical realm’s (theoretical and practical) problems from Faculty member, students and nurses viewpoint

**Variables**	**Faculty member (n=12)**	**Nursing student** **(n=60)**	**Nurses personal(n= 129)**	**Total(n=201)**	**p**
	**Agree ** **(N%)**	**Agree (N%)**	**Agree (N%)**	**Agree (N%)**	
Learnt theoretical subjects not coming into practice in clinical environment after graduation.	11(91.7)	47(78.3)	94(72.9)	152(75.6)	0.294
Weakness and usefulness of scientific function encouragement systems in clinic.	12(100)	42(70)	113(91.1)	167(85.2)	0.001
Lack of appropriate facilities in the ward in order of care based on nursing knowledge	10(83.3)	36(60)	79(62.2)	125(62.8)	0.303
Lack of caring standards based on nursing knowledge in the ward	6(50)	49(81.7)	89(70.1)	144(72.4)	0.002
Insufficient time for performance based on knowledge in connection with the nurse's workload	11(91.7)	47(78.3)	115(89.8)	173(86.5)	0.085
Match of the content of continuing education program with nurses' professional needs	4(33.3)	32(58.2)	51(40.5)	87(45.1)	0.062
Weakness and usefulness of scientific function encouragement systems in clinic.	12(100)	42(70)	113(91.1)	167(85.2)	0.001

* Chi-square test

## Discussion

Our aim in this study was to evaluate the educational and clinical problems to achieve competency by nurses in terms of academic staff, nursing students, and graduated nurses’ viewpoints. The findings of this study showed that the most important problem in nursing education was not applying the studies and research done in the clinical practice and this would led to non-evidence based training. Madarshahian et al. also showed that using evidence based training instead of traditional training would enhance knowledge, skill and high quality caring. As this an important factor in clinical competence, it ought to be included in nursing education (11).

Based on this study, another important factor influencing the clinical competence was the educators’ role. Cheraghi and Salsali, also reached the same result in their studies (12). Baraz et al. propounded presence of interested, and highly-experienced educators in the University of Medical Sciences as the most important strength of clinical teaching status (13). In Masoodi et al.’s study, the educators had referred to lack of a suitable environment, and lack of enough confidence for scientific discussions when necessary, as the two most prominent problems in their work (14).

Valizade et al. had also put forward the challenges facing the nursing students throughout their studies such as lack of integration of knowledge and practice, failure to apply the theory (theoretical knowledge) to practice, not using nursing process and scientific principles in clinical environment (15).

Ghodsbin and Shafakhah had also mentioned the students’ dissatisfaction with the staff-students rapport and the way of instructing the theoretical and practical courses as two other obstacles in teaching clinical skills. They add that that educators’ practical and educational experience, the provision of an environment for students’ to experience clinical skills and a sense of responsibility for educators are facilitating factors (16). This represents the clinical educators’ key role in teaching, achievement and promotion of nursing students to the level of clinical competence. Alavi, in his study on educators’ roles in clinical education, has mentioned factors such as educators’ presence in clinic as a source of reassurance for students, educator as an evaluator, relation establisher, experience provider, motivator, and supervisor, to be very important in learning,. An educator with these features is the effective educator (17). Michau has also paid attention to the relationship of clinical education and function as an effective factor in creating a theory and practice gap. In his study, he proposed that we should develop a strategy which based on that students could use complementary skills in their internship in order to enhance learning and practical skills (18). Another reason which affects the clinical competency is not teaching the theoretical subjects based on specific knowledge of nursing. Cheraghi’s findings are in compliance with ours (12).

According this study, educational problems in the clinical realm included weakness and inertia of the incentive academic performance in clinic systems, lack of standard care based on nursing knowledge on the ward and lack of appropriate facilities for care based on the nursing knowledge on the ward. Nemadi et al. have also reached similar results as the least supportive elements in the clinic from the nursing graduates such as lack of support from colleagues and administrators in improving knowledge and clinical skills (19). Mirzabeigi et al. showed that only a third of nurses are satisfied with their career. Job safety, satisfaction with work environment and facilities had the highest satisfaction level but the duties of the nursing profession, social status of the nursing profession and nursing procedures, and the communication with nursing managers had the highest dissatisfaction level (20).

Cheraghi also maintains that there is a mutual relationship between knowledge and practice in nursing. Paradoxes in nursing knowledge and nursing practice which are seen along with diverging organizational structure reduce the ability of nurses in using professional knowledge and nursing skills. These factors are effective in creating a gap between theory and practice (12). Maben et al., too, discovered the mismatch between theory and practice and its profound impacts on nurses’ psychological state, job satisfaction and its scientific records. He stated that nursing care should be holistic, patient-centred, based on nursing standards and research based (21).

## Conclusion

This cross-sectional study was done in an Iranian Medical University. So the results may not be generalized. This study investigated problems in achieving nursing competency in training programs from the participants’ viewpoint. The gap between the theory and practice was an important finding. Practical-orientation and traditional view of educators, students and nurses about clinical competency based on practical skills reduced the need of practice based on knowledge and research.  Efforts to reduce the gap between the theory- practice in educational and work environment are needed to improve training of qualified nurses. Also education of professionalism and action based on work environment may be useful. 
